# Protein-Energy Malnutrition Developing after Global Brain Ischemia Induces an Atypical Acute-Phase Response and Hinders Expression of GAP-43

**DOI:** 10.1371/journal.pone.0107570

**Published:** 2014-09-26

**Authors:** Shari E. Smith, Sarah A. Figley, David J. Schreyer, Phyllis G. Paterson

**Affiliations:** 1 College of Pharmacy and Nutrition, University of Saskatchewan, Saskatoon, Saskatchewan, Canada; 2 Department of Anatomy and Cell Biology, University of Saskatchewan, Saskatoon, Saskatchewan, Canada; 3 Cameco MS Neuroscience Research Center, Saskatoon City Hospital, Saskatoon, Saskatchewan, Canada; Indian Institute of Integrative Medicine, India

## Abstract

Protein-energy malnutrition (PEM) is a common post-stroke problem. PEM can independently induce a systemic acute-phase response, and pre-existing malnutrition can exacerbate neuroinflammation induced by brain ischemia. In contrast, the effects of PEM developing in the post-ischemic period have not been studied. Since excessive inflammation can impede brain remodeling, we investigated the effects of post-ischemic malnutrition on neuroinflammation, the acute-phase reaction, and neuroplasticity-related proteins. Male, Sprague-Dawley rats were exposed to global forebrain ischemia using the 2-vessel occlusion model or sham surgery. The sham rats were assigned to control diet (18% protein) on day 3 after surgery, whereas the rats exposed to global ischemia were assigned to either control diet or a low protein (PEM, 2% protein) diet. Post-ischemic PEM decreased growth associated protein-43, synaptophysin and synaptosomal-associated protein-25 immunofluorescence within the hippocampal CA3 mossy fiber terminals on day 21, whereas the glial response in the hippocampal CA1 and CA3 subregions was unaltered by PEM. No systemic acute-phase reaction attributable to global ischemia was detected in control diet-fed rats, as reflected by serum concentrations of alpha-2-macroglobulin, alpha-1-acid glycoprotein, haptoglobin, and albumin. Acute exposure to the PEM regimen after global brain ischemia caused an atypical acute-phase response. PEM decreased the serum concentrations of albumin and haptoglobin on day 5, with the decreases sustained to day 21. Serum alpha-2-macroglobulin concentrations were significantly higher in malnourished rats on day 21. This provides the first direct evidence that PEM developing after brain ischemia exerts wide-ranging effects on mechanisms important to stroke recovery.

## Introduction

Protein-energy malnutrition (PEM) is commonly associated with stroke. Pre-existing PEM is present in 12–19% of patients admitted to hospital with a diagnosis of stroke [1], [2], [3]. With post-stroke feeding challenges, PEM prevalence rises to 20–35% after one week [2],[4] and 35–49% by admission to a rehabilitation unit [5], [6]. These unfortunate statistics were first documented more than two decades ago and span many countries [7], [8].

In clinical studies, PEM is associated with poorer functional outcome following stroke [1], [2], [9], implicating malnutrition as an important stroke co-morbidity factor. Using a preclinical model, we found pre-existing PEM to be a direct cause of impaired short-term cognitive function after global brain ischemia [10]. While clinical studies have focused on the relationship between nutritional status and infection rates, length of hospital stay [8], and muscle strength [11], we identified direct effects on the ischemic brain. In rodent models of global brain ischemia mimicking the clinical scenario of PEM pre-existing at the time of ischemia and continuing untreated, PEM appeared to lower the set-point for the inflammatory response in the vulnerable hippocampal CA1 subregion [10], [12]. This was evident as an increase in activation of the predominant pro-inflammatory transcription factor, nuclear factor kappa B [12], and, in a subset of malnourished rodents, an augmented glial response to global brain ischemia [10]. While pre-existing PEM did not exacerbate neuronal death, neuroplasticity-related proteins were altered [10], [13]. The latter might be due to an increase in glial cell activation and neuroinflammation, which can modulate post-stroke neuroplasticity [14]. Whereas glial cells activated by brain ischemia can secrete growth factors [15], [16] that promote neuroplasticity, over-expression of pro-inflammatory cytokines and neuroinflammation can inhibit synaptic plasticity [17], [18].

No studies have addressed the response of the brain to the more common clinical problem of PEM developing after stroke. Thus, the first aim of the current study was to investigate the effects of post-ischemic PEM on glial activation, neuronal growth state, and synaptic organization. Since inflammation can persist for weeks to months after brain ischemia [19] when the brain is receptive to remodeling [20], we hypothesized that PEM would intensify the neuroinflammatory response, thus inhibiting the expression of growth associated protein-43 (GAP-43), synaptophysin and synaptosomal-associated protein-25 (SNAP-25).

PEM may also influence post-stroke recovery by altering the acute-phase reaction triggered by brain ischemia [21]. Although PEM can independently stimulate systemic inflammation [22], [23] and an atypical acute-phase response [24], malnutrition can also blunt such a response to injury or infection [25], [26]. Thus, the second objective was to investigate whether post-ischemic malnutrition would alter an acute-phase response induced by global ischemia. This was assessed by examining serum concentrations of the rat acute-phase proteins, albumin [27], alpha-2-macroglobulin (A2M), haptoglobin, and alpha-1-acid glycoprotein (AGP) [28].

## Materials and Methods

### Animals

Sixty-nine male Sprague-Dawley rats (52–55 day old) (Charles River Canada, QC, Canada) were acclimatized on rat chow for 2 days before placement on a protein adequate control diet (CON, 18% protein) [24]. Rats were housed in groups of 2–4 and maintained on a 12 hour light/dark cycle in a temperature controlled room with free access to food and water. This work was approved by the University of Saskatchewan's Animal Research Ethics Board and adhered to the Canadian Council on Animal Care guidelines for humane animal use.

### Global brain ischemia

Following a 4–6 day acclimation period on CON diet, rats were randomly allocated to undergo either sham surgery (Sham) or transient forebrain ischemia via the 2-vessel occlusion (2-VO) model (ISC), as previously described [29] and modified from Smith *et al*. [30]. Surgical procedures were performed aseptically under isoflurane anaesthesia (1.75–4% in 70% N_2_O and 30% O_2_). Before surgery, rats were fasted for ∼16 hours to achieve consistent blood glucose levels. Following anaesthesia induction, animals were placed on a heated water blanket and a tympanic probe was inserted to estimate brain temperature (IT-18 flexible probe; Physitemp Instruments Inc. NJ, USA). Tympanic temperature was maintained close to 37.5°C with the use of an overhead infrared lamp (250W) controlled by an automated feedback temperature controller (CN9500; Omega Engineering Inc., CT, USA). Tail artery cannulation allowed for continuous measurement of mean arterial blood pressure (PressureMAT PDKTP4-PCS; PendoTech, NJ, USA) and collection of arterial blood samples (100 µL) for measurement of blood gases, hematocrit, and glucose concentration. Samples for the latter were taken prior to and after the period of ischemia. Both common carotid arteries were isolated and the right jugular vein was cannulated. Blood was withdrawn via the jugular vein into a warmed heparinized syringe until blood pressure reached ∼35 mmHg. At this time, micro-aneurysm clips (S&T Vascular Clamps HD-S; Fine Science Tools, BC, Canada) were applied to both carotid arteries for 10 minutes. Blood pressure was recorded at 1 minute intervals and maintained at 35–40 mmHg throughout the occlusion period by withdrawing or infusing blood as needed. After releasing the clamps, blood was slowly re-infused and incisions were sutured. A dose of bupivacaine (2 mg/kg) was divided equally for injection around the 2 incision sites. Sham rats were treated identically except that carotid arteries were not occluded and hypotension was not induced. Post-surgical care included monitoring recovery from anaesthesia in the acute period as well as monitoring and documenting healing of incisions, physical appearance, grooming, and body weight for the entire experimental period. During the first 3 days of the post-surgical recovery period, food was available in the cages for ease of access. Rats were singly housed until post-surgical day 3, at which time the rats were placed back with their original cage mates.

### Diet assignment

At 3 days following surgery, rats previously exposed to global ischemia were randomly assigned to either a protein-deficient diet (PEM, 2% protein) or to continue on the CON diet (18% protein) (Dyets, Inc., PA, USA). All rats exposed to sham surgery were provided with CON diet. Diets were modified from the American Institute of Nutrition-93G diet [31]; the diet composition was previously described in detail [24]. Rats of this age fed a 2% protein diet voluntarily reduce food consumption, thereby causing a decrease in both energy and protein intake [24], [32]. Body weight and food intake were recorded on the day of surgery (day 0) and post-surgical days 1 and 2. Following diet assignment (day 3), food intake was recorded daily, and body weight was recorded weekly and on the final day of the experiment. The sample sizes for the six experimental groups generated were: CON-Sham5d (n = 9), CON-ISC5d (n = 13), PEM-ISC5d (n = 12), CON-Sham21d (n = 8), CON-ISC21d (n = 11), and PEM-ISC21d (n = 11).

### Histology and immunofluorescence

At 5 or 21 days post-surgery, rats were anaesthetized under isoflurane (5% in 100% O_2_) and blood samples were collected via cardiac puncture prior to euthanasia. Rats were then perfused transcardially with 0.9% saline followed by 4% paraformaldehyde. Intact heads were submerged into 4% paraformaldehyde and stored overnight at 4°C. Brains were removed and post-fixed in paraformaldehyde for an additional 24 hours. Following fixation, brains were submerged into a 20% sucrose solution and stored at 4°C for 3 days to prevent ice crystal formation and tissue cracking. Fixed brains were placed in plastic cryomolds and embedded in Optimal Cutting Temperature (OCT) compound. Samples were flash frozen using dry ice-cooled isopentane and stored at −80°C until sectioning. For each rat, the coronal section (14 µm) corresponding to −3.8 mm from Bregma was stained with cresyl violet, while adjacent sections were used for immunofluorescence. Viable CA1 and CA3 pyramidal neurons were counted at 400× magnification in cresyl violet stained sections. Using a 200 µm square (10×10) microscope grid, neurons with intact nucleus and cellular membrane were counted bilaterally in medial, middle, and lateral sectors of the CA1 region and the medial sector of the CA3 bend. Cell counts from the right and left hemisphere were summed to generate the total neuron count for each rat. Assessment of neuronal counts and semi-quantification of immunofluorescence were blinded.

Identical protocols were followed, using Shandon Sequenza Immunostaining Racks (Thermo Scientific, CHS, UK), to immuno-label coronal sections for astrocytes (GFAP), activated microglia (Iba-1), and the axon terminal proteins synaptophysin, SNAP-25, and GAP-43. Sections were blocked with normal goat serum (5%; Sigma-Aldrich, ON, Canada) and incubated at 4°C overnight in a primary antibody either for astrocytes (Rabbit anti-GFAP, 1∶400; Z0334, DakoCytomation, ON, Canada), microglia (Rabbit anti-Iba-1, 1∶1000; 019–19741, Wako Chemicals, VA, USA), synaptophysin (Mouse anti-synaptophysin, 1∶200; clone SY38, Millipore, MA, USA), SNAP-25 (Mouse anti-SNAP-25, 1∶500; Millipore, clone SP12, MA, USA) or GAP-43 (Mouse anti-GAP-43, 1∶2500; clone 9-1E12 Ascites fluid [33]). Following a wash step with PBS, slides were incubated in a secondary fluorescent antibody (Goat-anti-mouse AlexaFluor488, 1∶200 [115-545-166, Jackson Laboratories, PA, USA] or Goat-anti-rabbit AlexaFluor 594, 1∶500 [A11012, Molecular Probes, Invitrogen]) in the dark for 2 hours. Slides were washed with PBS and cover slipped with ProLong Gold Antifade Reagent with DAPI (P36931, Molecular Probes, Invitrogen) in order to visualize cell nuclei. Negative control sections were treated identically, except that the sections were not incubated in the primary antibody.

Semi-quantification of the immuno-labeling obtained for each marker was completed using one section per hemisphere from every rat (ImageJ, U.S. National Institutes of Health, MD, USA). The integrated density value (IDV), the sum of the pixel values in the region of interest, was measured using high magnification photographs captured from a fluorescence microscope. All densitometry measurements were adjusted for background staining, as calculated from negative control sections. GFAP and Iba-1 expression was measured by placing a box (pixel area  = 265,816) that included the CA1 pyramidal layer, stratum oriens and stratum radiatum. A second box was placed in the CA3 mossy fiber terminals (pixel area  = 265,816). Immuno-staining of axon terminal markers was found predominantly within the CA3 region, and therefore semi-quantification was performed in the mossy fiber terminals for synaptophysin (pixel area  = 78,402), SNAP-25 (pixel area  = 101,936), and GAP-43 (pixel area  = 141,778). Values from the right and left hemispheres were averaged.

### Serum acute-phase proteins

Blood samples collected at the time of euthanasia were allowed to clot for 30 minutes at room temperature and then centrifuged at 1,500×g for 10 minutes. Following centrifugation, serum was collected and stored at −80°C. Enzyme-linked immunosorbent assay (ELISA) kits were used to measure the serum concentrations of A2M, AGP, and haptoglobin (Immunology Consultants Laboratory, Inc., OR, USA), which are positive acute-phase proteins in the rat [28]. Serum concentrations of the negative acute-phase protein, albumin [27], was measured using the bromocresol green method [34]. A volume of 25 µL of blank, serum sample, or standard solution was added to 5.0 mL bromocresol green reagent (0.15 mmol/L bromocresol green, 0.075 mol/L succinate buffer, 30% Brij-35). After 30 minutes, absorbance was measured spectrophotometrically at 628 nm (Biochrom Ultrospec 3100 Pro). Linear regression was used to determine the albumin concentration of the samples.

### Statistical analyses

Statistical analyses were conducted using SPSS 20.0 for Windows. All data are presented as mean ± SEM, and the significance level was set at p<0.05. Body weight and food intake differences between rats exposed to sham surgery or global ischemia were analyzed on post-surgical days 0, 1, 2, and 3 using an independent-sample Student's t-test. Following assignment to experimental diet on day 3, food intake was analyzed for rats fed the low protein diet or CON diet for each day until it reached significance using an independent-sample Student's t-test. Significant differences in body weight were determined using a 1-factor ANOVA and Tukey's test. Differences in neuronal cell counts, serum acute-phase protein concentrations, and immunofluorescence among the three treatment groups at each time-point were determined using a 1-factor ANOVA and Tukey's test for two *posthoc* comparisons only; comparing the CON-Sham and the CON-ISC groups identified an ischemia effect, whereas the CON-ISC versus PEM-ISC comparison detected an effect of diet on ischemic outcome. Selected unadjusted pair-wise comparisons were also made for the immunofluorescence data.

## Results

### Exclusion from the study

Five rats were excluded from the study due to blood pressure readings not meeting criteria, unsuccessful tail artery cannulation, or poor recovery demonstrated by severe weight loss. These rats were humanely euthanized with isoflurane (5% in 100% O_2_).

### Physiological parameters measured during surgery

A summary of the physiological parameters measured during 2-VO and sham surgeries in the remaining rats is shown in **Table S1**. Although all rats were still on CON diet at the time of surgery, the table separates the CON-ISC and PEM-ISC groups to illustrate baseline comparison prior to dietary assignment. Mean pre- and post-ischemic values for all physiological parameters were within the target range. Tympanic temperature was tightly regulated throughout the 10 minute occlusion period, and thus the resulting tympanic temperature values fell within the desired range for the three experimental groups. Blood pressure was carefully manipulated in the two groups exposed to global ischemia, resulting in values within the desired range.

### Impact of surgery on food intake and body weight prior to diet assignment

Mean initial body weight on the day of surgery was not significantly different between sham and ischemic treatment groups, at which time all rats were on CON diet (t (62) = −0.38, p = 0.71; Sham, n = 17; ISC, n = 47). A significant decrease in body weight was reported on post-surgical days 1, 2 and 3 in the group exposed to the 2-VO surgery, relative to that of sham animals (day 1, t (62) = 5.23, p<0.001; data not shown). Individual rat food intake was monitored on post-surgical days 1, 2, and 3, which was possible due to individual rat housing (Sham, n = 17; ISC, n = 47). Daily food intake was significantly decreased in the ischemia treatment group compared to sham-operated rats on days 1, 2 and 3 (day 1, t (62) = 13.29, p<0.001; data not shown).

### Indices of PEM

**Figure 1:**
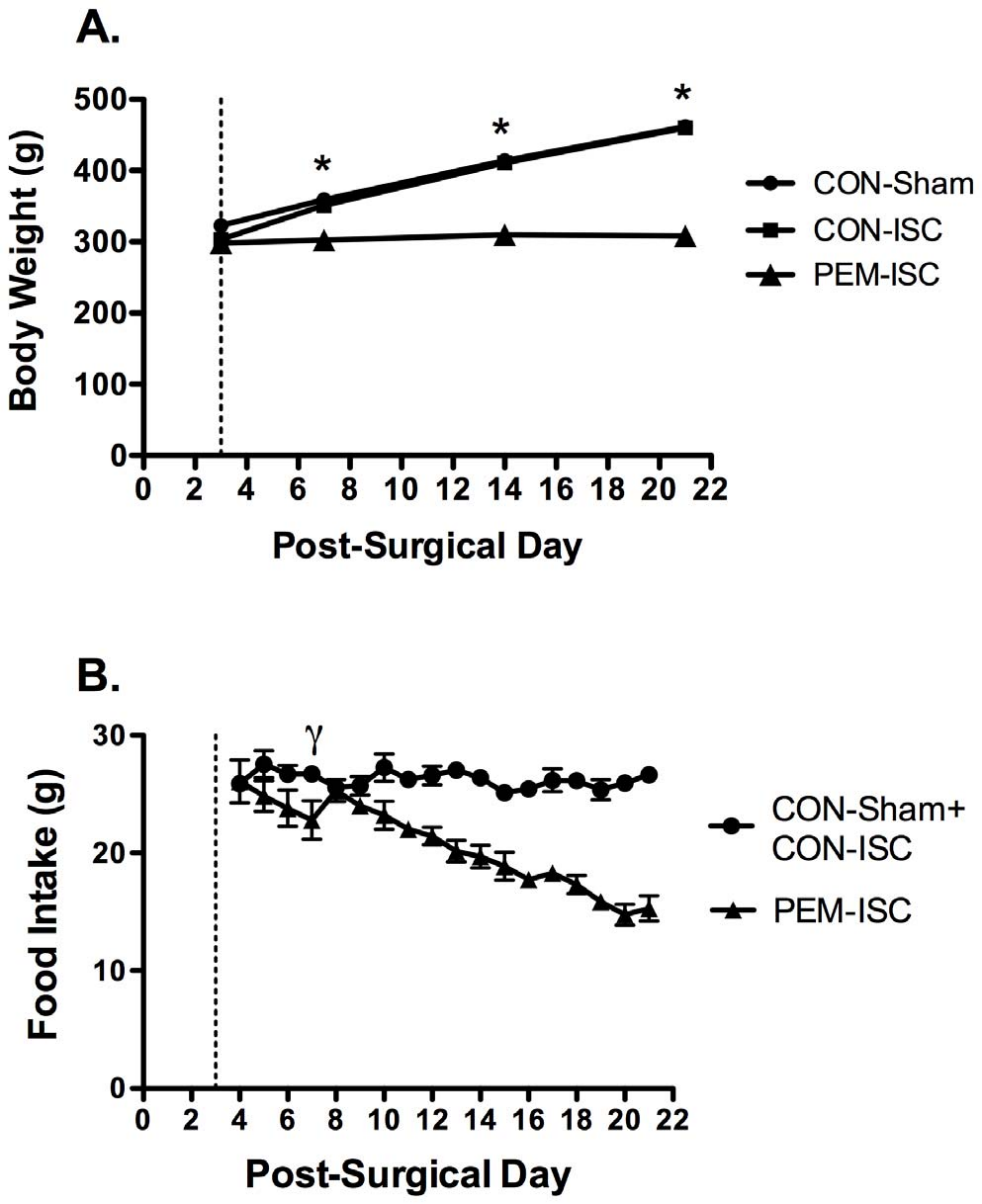
The PEM regimen introduced on day 3 after global brain ischemia depressed body weight and food intake. Data are shown as mean ± SEM for the day 21 treatment groups. The dashed vertical line illustrates the day on which rats were assigned to experimental diet. (**A**) Body weights are shown for days 3, 7, 14 and 21 (CON-Sham21d, n = 8; CON-ISC21d, n = 11; PEM-ISC21d, n = 11).*Indicates a significant effect of experimental diet on body weight (PEM-ISC compared to CON-Sham and CON-ISC groups) by Tukey's Test (p<0.05). (**B**) Food intake was collected daily on a cage basis (CON-Sham21d + CON-ISC21d, n = 8 cages [2–3 rats/cage]; PEM-ISC21d, n = 5 cages [2–3 rats/cage]) and calculated as daily cage food intake/number of rats per cage. γ Indicates the first day on which PEM-ISC rats experienced a significant reduction in food intake, when compared to that for the combined CON groups, as detected by an independent-sample Student's t-test (p<0.05).

**Fig. 1A** shows the pattern of body weight change for the day 21 treatment groups resulting from experimental diet assignment initiated on post-surgical day 3. On day 7 post-surgery (4 days after diet assignment), there was a significant difference in body weight (F_2,27_ = 31.74, p<0.001), with PEM-ISC rats weighing significantly less than CON-Sham (Tukey's test; p<0.001) and CON-ISC (p<0.001) rats; this pattern continued throughout the post-surgical period (p<0.001). Malnourished rats gained 6% of their initial body weight by day 21, compared to a 59% increase observed in both the CON-ISC and CON-Sham groups. This resulted in a mean difference in final body weight of 33% between the PEM-ISC group and both CON-fed groups.

**Fig. 1B** shows mean (± SEM) daily food intake starting on post-surgical day 4 (1 day after diet assignment) for rats fed the low protein (PEM-ISC21d, n = 5 cages [2–3 rats/cage]) or CON diet (CON-Sham21d + CON-ISC21d, n = 8 cages [2–3 rats/cage]). Mean food intake was calculated based on cage data (daily cage food intake/number of rats per cage). Since CON-ISC and CON-Sham rats were sometimes housed in the same cages, differences between these groups could not be analyzed. A significant decrease in food intake in the PEM-ISC group was first observed on post-surgical day 7 (4 days after diet assignment), as compared to the other two CON groups combined (t (11) = 2.57, p = 0.026). Total food intake over the 18 days on experimental diet was depressed by 21% in PEM-ISC rats. Rats in the day 5 treatment groups had similar body weight and food intake patterns to the day 21 groups (data not shown).

### CA1 and CA3 neuronal death

Hippocampal CA1 neuronal counts are shown in **Table 1**. On both days 5 and 21 post-ischemia, there was a significant group effect (5 day, F_2,24_ = 217.41, p<0.001; 21 day, F_2,22_ = 296.29, p<0.001). There was extensive CA1 neuronal death in the CON-ISC rats compared to CON-Sham rats (Tukey's test; p<0.001), but no significant difference between the PEM-ISC and CON-ISC groups (p>0.87). Hippocampal CA3 neuronal counts did not differ among treatment groups at post-surgery day 21 (F_2,21_ = 2.41, p = 0.114). Mean (± SEM) counts were 77±3 (CON-Sham; n = 8), 72±3 (CON-ISC; n = 10), and 81±2 (PEM-ISC; n = 8). CA3 neurons were counted to verify that the differences in axon terminal marker proteins within the CA3 mossy fibers between PEM-ISC and CON-ISC groups on day 21 were not related to CA3 neuronal loss.

**Table 1:** PEM initiated at 3 days after global brain ischemia did not exacerbate hippocampal CA1 neuronal death.

	CON-Sham	CON-ISC	PEM-ISC
Day 5	258±10 (n = 8)	46±7 (n = 11)^*^	41±7 (n = 8)
Day 21	241±10 (n = 8)	27±7 (n = 7)^*^	22±4 (n = 10)

Data are presented as mean ± SEM. *CA1 neuronal counts were significantly decreased in the CON-ISC group, as compared to the CON-Sham group by 1- factor ANOVA and Tukey's test (p<0.001), but the CON-ISC and PEM-ISC groups did not differ (p>0.87).

The 2-VO model of global brain ischemia yields some variability in CA1 neuronal death, and thus the statistical analyses for the neuron counts in Table 1 excluded those rats exposed to 2-VO surgery that showed CA1 neuronal death that was unilateral or minimal (<50% reduction in CA1 neurons in either hemisphere). This was done only after first verifying that there were no significant differences in neuronal loss between the PEM-ISC and CON-ISC groups. That is, it was first demonstrated that excluding rats with incomplete forebrain ischemia did not bias the results by masking an effect of PEM on neuronal death at either time-point (day 5, t (23) = −1.35, p = 0.16).

Analyses of the other endpoints (below) were conducted only on rats with extensive bilateral hippocampal damage on day 5 (CON-Sham, n = 8; CON-ISC, n = 11; PEM-ISC, n = 8) and day 21 (CON-Sham, n = 8; CON-ISC, n = 7; PEM-ISC, n = 10). Exceptions to this sample size were related to instances in which blood samples were unattainable or tissue quality prevented immunofluorescence analysis (Iba-1, CON-ISC21d, n = 6; GAP-43, PEM-ISC21d, n = 9; SNAP-25, CON-ISC5d, n = 9).

### Glial response to global brain ischemia

**Figure 2:**
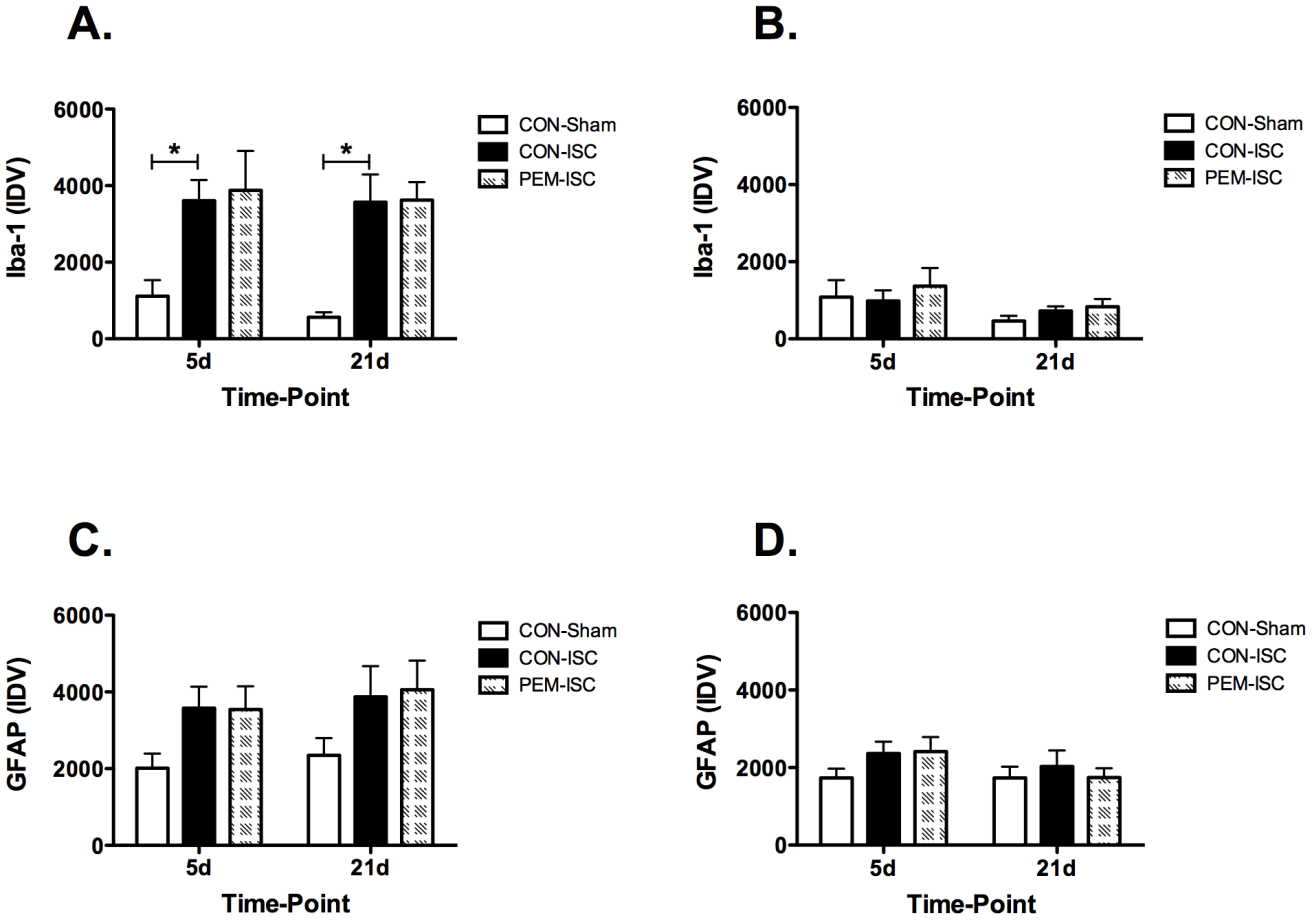
PEM introduced on day 3 after global ischemia did not alter the hippocampal glial response on either day 5 or 21. Immuno-labeling results are shown for Iba-1 in the CA1 (A) and CA3 (B) and for GFAP in the CA1 (C) and CA3 (D) hippocampal subregions.*Indicates a significant difference for the *posthoc* comparisons made by Tukey's Test (CON-Sham versus CON-ISC) within the specific time-point (p<0.05). Results are shown as mean ± SEM integrated density value (IDV).

**Figure 3:**
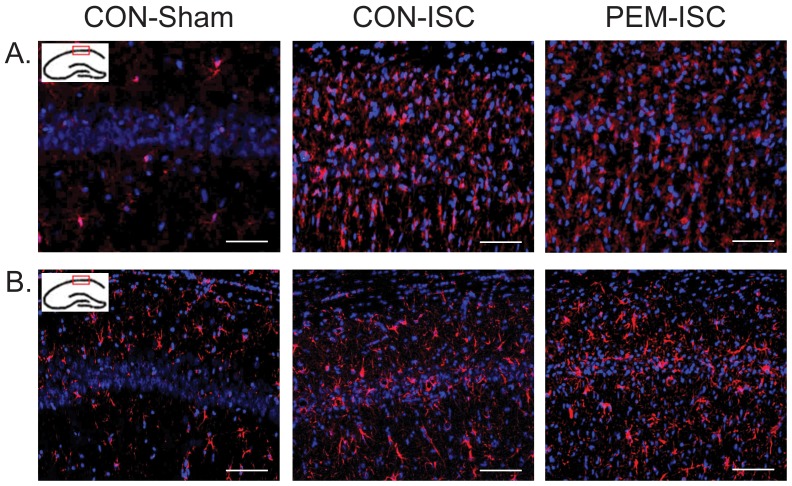
Representative photographs of the Iba-1 (A) and GFAP (B) immunofluorescence in the CA1 hippocampal subregion on day 21 after global brain ischemia. Blue  =  DAPI (cell nuclei). Red  =  Iba-1 (activated microglia marker; A) or GFAP (astrocytic marker; B).

Semi-quantification of Iba-1 immunofluorescence is shown for the CA1 (**Fig. 2A**) and CA3 (**Fig. 2B**) hippocampal subregions. Within the CA1 subregion at both time-points, there was a significant group effect on Iba-1 levels (day 5, F_2,24_ = 4.55, p = 0.021; day 21, F_2,24_ = 12.85, p<0.001). Iba-1 staining was significantly increased by ischemia in the control-fed rats (CON-Sham vs CON-ISC by Tukey's test; day 5, p = 0.040; day 21, p = 0.001), but this was not influenced by post-ischemic PEM at either time-point (CON-ISC vs PEM-ISC; day 5, p = 0.957; day 21, p = 0.997). A representative photograph of Iba-1 staining in the CA1 subregion on day 21 is shown in **Fig. 3A** for each experimental group. Within the CA3 subregion, Iba-1 staining was not as pronounced, and there was no significant effect of experimental group at either time-point (day 5, F_2,23_ = 0.26, p = 0.771; day 21, F_2,21_ = 1.30, p = 0.294).

GFAP expression analysis is also presented for the hippocampal CA1 (**Fig. 2C**) and CA3 (**Fig. 2D**) subregions. There were no group effects on the GFAP signal that reached statistical significance for either time-point within the CA1 (day 5, F_2,24_ = 2.61, p = 0.095; day 21, F_2,22_ = 1.79, p = 0.191) or CA3 (day 5, F_2,24_ = 1.35, p = 0.278; day 21, F_2,22_ = 0.27, p = 0.769) subregions. A representative photograph of GFAP staining in the CA1 subregion on day 21 is shown in **Fig. 3B**.

### Axon terminal markers following global brain ischemia

**Figure 4:**
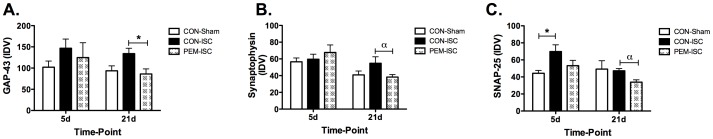
The influence of post-ischemic PEM on expression of GAP-43 (A), synaptophysin (B) and SNAP-25 (C) within the CA3 mossy fibers on days 5 and 21 following global brain ischemia. *Indicates a significant difference for either of the 2 *posthoc* comparisons made by Tukey's Test (CON-Sham versus CON-ISC and CON-ISC versus PEM-ISC) within the specific time-point (p<0.05). α Indicates a significant difference between CON-ISC and PEM-ISC groups detected by unadjusted pairwise comparison (p<0.05). Results are shown as mean ± SEM integrated density value (IDV).

Semi-quantification of GAP-43, synaptophysin, and SNAP-25 within the CA3 mossy fiber terminals is shown in **Fig. 4A, 4B, and 4C**, respectively. There was no effect of treatment on GAP-43 expression at day 5 (F_2,24_ = 1.13, p = 0.339). On post-ischemic day 21, the group effect was significant (F_2,21_ = 4.23, p = 0.029), with GAP-43 decreased in the PEM-ISC group as compared to that in the CON-ISC rats (Tukey's test; p = 0.030). A trend was present for GAP-43 immuno-staining to increase in the CON-ISC group at post-ischemic day 21 as compared to that in the CON-Sham rats (Tukey's test; p = 0.082).

For synaptophysin expression, there was no significant effect of treatment on post-ischemic day 5 (F_2,24_ = 0.62, p = 0.548). On day 21, the group effect was not statistically significant although a trend was evident (F_2,22_ = 2.91, p = 0.076). An unadjusted pairwise comparison demonstrated a significant depression in the synaptophysin signal in the PEM-ISC group as compared to that in the CON-ISC rats (t (15) = 2.23, p = 0.041).

There was a significant group effect on SNAP-25 expression on day 5 (F_2,22_ = 4.36, p = 0.025), with a marked increase due to ischemia in the control-fed rats (CON-Sham vs CON-ISC by Tukey's test; p = 0.022). PEM had no effect on the ischemic response at day 5 (CON-ISC vs PEM-ISC; p = 0.169). Although the 1-factor ANOVA did not reveal a significant group effect at post-ischemic day 21 (F_2,22_ = 2.17, p = 0.138), an unadjusted pairwise comparison demonstrated a significant decrease in SNAP-25 in the PEM-ISC group as compared to that in the CON-ISC rats (t (15) = 3.38, p = 0.004).

**Figure 5:**
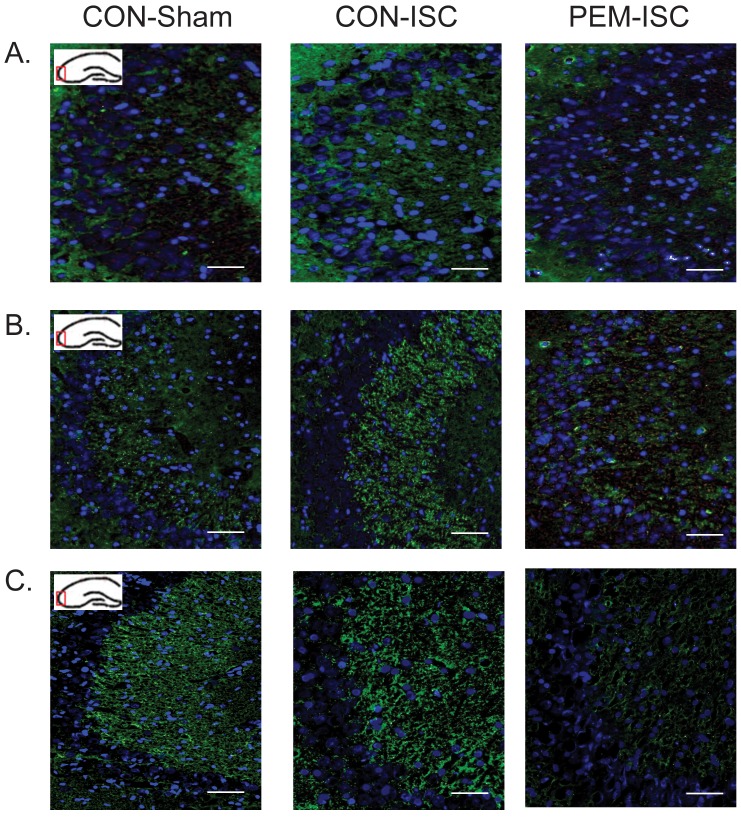
Representative photographs of GAP-43 (A), synaptophysin (B) and SNAP-25 (C) immunofluorescence in the CA3 mossy fibers on day 21 after global brain ischemia. Blue  =  DAPI (cell nuclei). Green  =  GAP-43 (A), synaptophysin (B), or SNAP-25 (C).

Representative photographs of the CA3 mossy fiber region for GAP-43 (**Fig. 5A**), synaptophysin (**Fig. 5B**) and SNAP-25 (**Fig. 5C**) are shown for post-ischemic day 21.

### Acute-phase response after global brain ischemia

**Figure 6:**
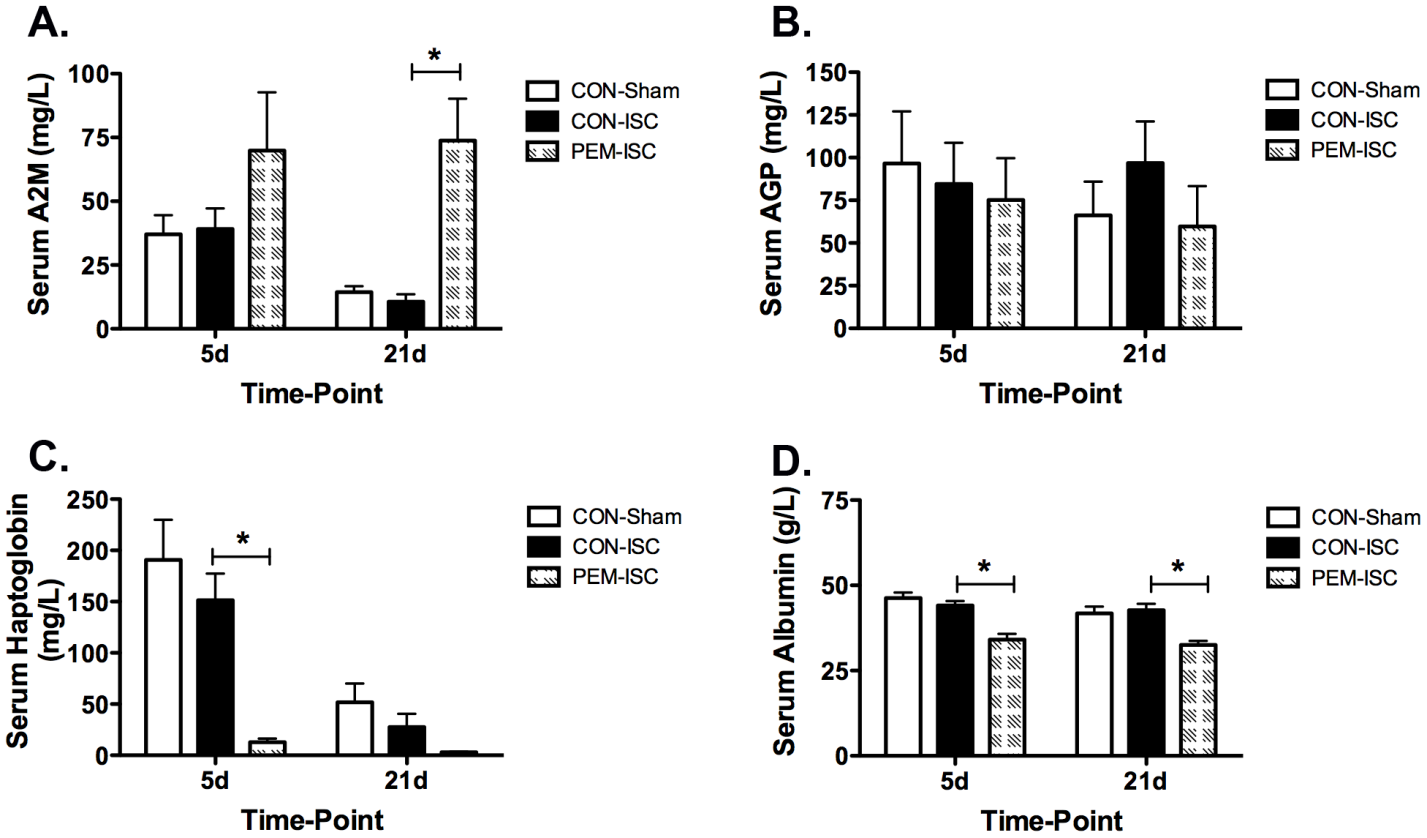
PEM introduced on day 3 following global brain ischemia elicits an atypical acute-phase response. Alpha-2-macroglobulin (A2M) (**A**), alpha-1-acid glycoprotein (AGP) (**B**), and haptoglobin (**C**) are positive acute-phase proteins, and albumin (**D**) is a negative acute-phase protein. *Indicates a significant difference for the 2 *posthoc* comparison made by Tukey's Test (CON-ISC versus PEM-ISC) within the specific time-point (p<0.05). Data are shown as mean ± SEM for each experimental group on day 5 (CON-Sham, n = 8; CON-ISC, n = 11; PEM-ISC, n = 6) and day 21 (CON-Sham, n = 8; CON-ISC, n = 6; PEM-ISC, n = 10).

**Fig. 6** shows the serum concentrations of positive (A2M, AGP, and haptoglobin) and negative (albumin) acute-phase proteins for post-surgery days 5 and 21. Serum A2M concentrations were not significantly different among the three treatment groups on day 5 (F_2,22_ = 1.96, p = 0.165). A significant group effect was evident by day 21 (F_2,21_ = 9.27, p = 0.001), with significantly higher circulating levels of A2M in the PEM-ISC group as compared to those of CON-ISC rats (Tukey's test; p = 0.005). Serum haptoglobin concentrations were significantly different among treatment groups at both time-points (day 5, F_2,22_ = 8.00, p = 0.002; day 21, F_2,21_ = 4.67, p = 0.021). The PEM-ISC group had a decrease in haptoglobin levels, relative to CON-ISC rats, that was statistically significant on day 5 (Tukey's test; p = 0.011), but not day 21 (p = 0.353). Serum albumin showed a significant group effect at both day 5 (F_2,22_ = 15.49, p<0.001) and day 21 (F_2,21_ = 12.38, p<0.001), with concentrations decreased in the PEM-ISC group as compared to the CON-ISC group (Tukey's test; day 5, p<0.001; day 21, p = 0.001). There was no effect of treatment on serum AGP concentrations at either time-point (day 5, F_2,22_ = 0.13, p = 0.88; day 21, F_2,21_ = 0.64, p = 0.54).

There were no significant differences between the CON-ISC and CON-Sham groups for any acute-phase protein at either time-point (Tukey's test; p>0.40).

## Discussion

Suboptimal protein and energy status is a common problem arising in patients after and because of a stroke. Here, we report on the first study to utilize a well-controlled experimental model to isolate the direct effects of this compromised nutritional state on the post-ischemic brain. Specifically, we found that PEM developing after global brain ischemia inhibits the expression of axon terminal proteins that serve as markers for synaptic remodeling in the hippocampal CA3 mossy fibers. In addition, the induction of PEM elicited a strong systemic response characterized by an immediate and sustained atypical acute-phase reaction. These findings highlight important mechanisms by which malnutrition developing in the post-stroke period could hamper recovery and suggest that treating this co-morbidity factor may be essential for patients to benefit fully from rehabilitation therapy.

To address the specific effects of post-ischemic PEM on the brain, we utilized a well-controlled and previously characterized nutritional regimen in which a low protein diet causes a voluntary reduction in food intake and induces mixed PEM [24]. Introducing this regimen on the third post-ischemic day provided evidence that PEM could interfere with axonal sprouting elicited by brain ischemia. We semi-quantified the SNAP-25, GAP-43 and synaptophysin immunofluorescence since these indicators of synaptic structure have previously been used to evaluate neuroplasticity in other models of cerebral ischemia [13], [35], [36], [37]. Synaptophysin and SNAP-25 are pre-axonal synaptic proteins that are often used to identify functional synaptic connections [38], [39]. GAP-43 is expressed in actively sprouting neurons, and specifically in the growth cones of axons [40], [41]. The GAP-43 expression showed clearly that by day 21, PEM can significantly hinder the potential for synaptic remodeling that is induced by global ischemia. Although less studied in global brain ischemia, focal (cortical) ischemia induces GAP-43 as part of its program to reactivate the growth potential of neurons in the region of axonal sprouting in the peri-infarct cortex [42]. We acknowledge that further studies are required to provide definitive evidence that PEM inhibits mossy fiber sprouting, since the decreases in synaptophysin and SNAP-25 on day 21 in the PEM-ISC group relative to the CON-ISC rats were not statistically significant by 1-factor ANOVA. Nonetheless, it is important to highlight the significant depression in the signals for these molecules detected by the unadjusted pairwise comparisons between the PEM-ISC and CON-ISC rats, particularly because semi-quantitative densitometry limits statistical power for detecting differences. The consistent inhibitory effect of post-ischemic PEM on all three synaptic markers on day 21 is evident in Fig. 4 and 5. Future studies with Golgi-Cox staining and electrophysiology to examine dendritic morphology and synaptic strength, respectively, will also be needed to reinforce the study results. Our findings are reminiscent of the extensive literature on the adverse effects of perinatal malnutrition on synaptic plasticity in the hippocampal formation [43], [44], although some of these studies are limited by flaws in the methodology for the nutritional regimens (reviewed in [45]).

Depending on stroke severity, some spontaneous neurological recovery can occur that can be further enhanced by rehabilitation [46], [47]. Because of the link between increased plasticity and improved function after stroke [20], our results suggest that preventing or reversing the common post-stroke problem of PEM [2], [4], [6] could directly enhance spontaneous recovery and facilitate rehabilitation efforts aimed at enhancing plasticity [48]. While the global brain ischemia model mimicking cardiac arrest has been a valuable way to examine effects of post-ischemic PEM, these findings should be extended to establish functional relevance in an experimental model of focal ischemia mimicking the ischemic stroke patient.

The malnutrition-induced decrease in hippocampal synaptic remodeling is not caused by a different degree of brain injury, since the extent of neuronal death in the vulnerable hippocampal CA1 subregion did not differ between CON-ISC and PEM-ISC groups. Neither did PEM extend neuronal death into the more resistant CA3 subregion. These findings were expected, since the malnutrition was introduced after the period of major CA1 neuronal death [49]. The trigger for the reduced receptivity to brain remodeling also does not appear to be increased neuroinflammation. Microglia and astrocytes are predominant responders and the major inflammatory cells elicited by global brain ischemia [50]. Since the hippocampal CA1 glial response observed at post-ischemic days 5 and 21 in our study was unaffected by nutritional status, we found no evidence that PEM arising after global brain ischemia exacerbates brain inflammation. Future investigation of the balance between specific pro- and anti-inflammatory mediators would further strengthen this conclusion.

The second major finding of our study was that post-ischemic PEM triggered an atypical acute-phase response characterized by immediate and prolonged changes to specific circulating acute-phase proteins relative to the CON-ISC group. The decrease in the negative acute-phase protein, albumin, on day 2 after nutritional intervention (5 days following global ischemia) was sustained at day 21, at which time the rise in the class 2 positive acute-phase protein, A2M, also became significant. Our previous findings suggest that this reaction exists without a febrile response [24], [29]. The acute-phase response was selective, however, as the class 1 acute-phase protein, AGP, was unchanged, and haptoglobin was decreased after 2 days of exposure to the PEM regimen and barely detectable by day 21. The decrease in haptoglobin, a positive acute-phase protein, suggests that post-ischemic PEM induces an aberrant acute-phase reaction.

The implications to stroke recovery of an incomplete acute-phase reaction accompanying post-ischemic PEM should be further explored. A heightened acute-phase response in stroke patients is associated with worse outcome, although it is unclear whether it plays a direct detrimental role [21], [51], [52]. Prolonged systemic inflammation after focal ischemia can increase brain injury and be more detrimental than pre-existing inflammation [53]. Although PEM did not increase neuronal death, the malnutrition-induced decrease in expression of axon terminal proteins could be related to increased susceptibility of the malnourished, injured post-stroke brain to the systemic inflammation. Decreased long-term potentiation in the hippocampus occurs with systemic inflammation [54], [55], although it is of note that the systemic inflammation caused by PEM did not increase microglial activation as was previously reported [54]. Cognitive testing and serum cytokine measurements in future studies will help to address this hypothesis. Individual differences in nutritional status should also be investigated as a possible contributor to the considerable variability in the acute-phase response observed among stroke patients [21], [51].

Unfortunately, we were unable to completely unravel the potentially complex interactions among brain ischemia, acute-phase response, and PEM, since no acute-phase reaction was detected after global ischemia in the control diet-fed rats. That is, no differences existed between the CON-ISC and CON-Sham groups for any acute-phase protein at either time-point. Global brain ischemia, unlike focal ischemia, may not trigger an acute-phase reaction. Alternatively, the latter may not have been detectable above the much larger response typically elicited by surgery [56]. The relatively higher serum A2M and haptoglobin concentrations in both CON-Sham and CON-ISC rats on day 5 relative to day 21 is suggestive of a surgery-induced acute-phase response that subsides over time. This raises a potential limitation in using the currently available rat models of brain ischemia to study the role of the acute-phase response as a determinant of stroke outcome, since the major models rely on surgery to generate reproducible brain ischemia. The least surgically invasive model should be chosen if the goal is to study the systemic reaction to brain ischemia.

In summary, the initiation of PEM within days of global brain ischemia triggers an immediate aberrant acute-phase response that is sustained. Post-ischemic PEM also hinders neuroplasticity mechanisms, which do not appear to be triggered by an increase in neuroinflammation. This is the first well-controlled study to demonstrate that post-ischemic PEM exerts direct and wide-ranging effects on mechanisms important to stroke recovery.

## Supporting Information

Table S1 **Physiological parameters measured during 2-VO and sham surgeries.**
(DOC)
