# Supplementary material for: Protein-Energy Malnutrition Developing after Global Brain Ischemia Induces an Atypical Acute-Phase Response and Hinders Expression of GAP-43
Source: PLoS One. 2014 Sep 26;9(9):e107570. doi: 10.1371/journal.pone.0107570 (PMC4178032; doi:10.1371/journal.pone.0107570)
Supplement: Table S1 — Physiological parameters measured during 2-VO and sham surgeries. (DOC) [file pone.0107570.s001.doc]

**Supplemental TABLE 1**. Physiological parameters measured during 2-VO and sham surgeries

|  | **CON-Sham** | **CON-ISC** | **PEM-ISC** |
| --- | --- | --- | --- |
| **Pre-Ischemic**  pH | 7.43 ± 0.01 | 7.42 ± 0.01 | 7.42 ± 0.01 |
| pCO2 (mmHg) | 35 ± 1 | 38 ± 1 | 37 ± 2 |
| pO2 (mmHg) | 125 ± 2 | 130 ± 2 | 130 ± 2 |
| Hct (%) | 37.8 ± 0.7 | 38.1 ± 0.6 | 40.0 ± 0.7 |
| Glucose (mmol/L) | 5.4 ± 0.2 | 5.6 ± 0.2 | 5.6 ± 0.2 |
| **Post-Ischemic**  pH | 7.42 ± 0.01 | 7.37 ± 0.01 | 7.33 ± 0.01 |
| pCO2 (mmHg) | 36 ± 1 | 38 ± 1 | 42 ± 2 |
| pO2 (mmHg) | 131 ± 3 | 135 ± 3 | 135 ± 3 |
| Hct (%) | 38.9 ± 0.9 | 35.0 ± 0.8 | 36.1 ± 0.9 |
| Glucose (mmol/L) | 5.5 ± 0.1 | 5.5 ± 0.2 | 5.1 ± 0.2 |
| **Intra-Ischemic**  Tympanic Temperature (°C)  Blood Pressure (mmHg) | 37.5 ± 0.01  86 ± 3 | 37.3 ± 0.01  36 ± 0.1 | 37.4 ± 0.02  35 ± 0.1 |

Data are expressed as mean ± SEM (CON-Sham, n=17; CON-ISC, n=24; PEM-ISC, n=23). Mean pre- and post-ischemic values for all measured physiological parameters fell within the desired range for all surgical groups.
